# microRNA‐146a controls age‐related bone loss

**DOI:** 10.1111/acel.13244

**Published:** 2020-10-21

**Authors:** Victoria Saferding, Melanie Hofmann, Julia S. Brunner, Birgit Niederreiter, Melanie Timmen, Nathaniel Magilnick, Silvia Hayer, Gerwin Heller, Günter Steiner, Richard Stange, Mark Boldin, Gernot Schabbauer, Moritz Weigl, Matthias Hackl, Johannes Grillari, Josef S. Smolen, Stephan Blüml

**Affiliations:** ^1^ Department of Rheumatology Medical University of Vienna Vienna Austria; ^2^ Ludwig Boltzmann Institute for Arthritis and Rehabilitation Vienna Austria; ^3^ Institute for Vascular Biology Centre for Physiology and Pharmacology Medical University of Vienna Vienna Austria; ^4^ Department of Regenerative Musculoskeletal Medicine Institute of Musculoskeletal Medicine (IMM) University Hospital Münster Münster Germany; ^5^ Department of Molecular and Cellular Biology Beckman Research Institute City of Hope Duarte California USA; ^6^ Department of Medicine I Medical University of Vienna Vienna Austria; ^7^ TAmiRNA GmbH Vienna Austria; ^8^ Austrian Cluster for Tissue Regeneration Vienna Austria; ^9^ Department of Biotechnology Institute for Molecular Biotechnology BOKU – University of Natural Resources and Life Sciences Vienna Austria; ^10^ Ludwig Boltzmann Institute for Experimental and Clinical Traumatology in AUVA Research Center Vienna Austria

**Keywords:** aging, bone metabolism, microRNA, osteopetrosis, osteoporosis

## Abstract

Bone loss is one of the consequences of aging, leading to diseases such as osteoporosis and increased susceptibility to fragility fractures and therefore considerable morbidity and mortality in humans. Here, we identify microRNA‐146a (miR‐146a) as an essential epigenetic switch controlling bone loss with age. Mice deficient in miR‐146a show regular development of their skeleton. However, while WT mice start to lose bone with age, animals deficient in miR‐146a continue to accrue bone throughout their life span. Increased bone mass is due to increased generation and activity of osteoblasts in miR‐146a‐deficient mice as a result of sustained activation of bone anabolic Wnt signaling during aging. Deregulation of the miR‐146a target genes Wnt1 and Wnt5a parallels bone accrual and osteoblast generation, which is accompanied by reduced development of bone marrow adiposity. Furthermore, miR‐146a‐deficient mice are protected from ovariectomy‐induced bone loss. In humans, the levels of miR‐146a are increased in patients suffering fragility fractures in comparison with those who do not. These data identify miR‐146a as a crucial epigenetic temporal regulator which essentially controls bone homeostasis during aging by regulating bone anabolic Wnt signaling. Therefore, miR‐146a might be a powerful therapeutic target to prevent age‐related bone dysfunctions such as the development of bone marrow adiposity and osteoporosis.

## Graphical abstract

We identify microRNA‐146a as a molecular clock that controls bone loss during aging by restricting bone anabolic pathways and promoting bone marrow adiposity. Loss of miR‐146a preserves bone anabolic pathways, leading to continuous increases in bone mass during aging, and protects from the development of experimental osteoporosis. Therefore, we identify miR‐146a as an essential factor regulating bone loss and suggest that targeting miR‐146a might be a powerful means to treat bone loss in diseases such as osteoporosis.

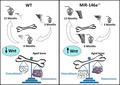

## 1 INTRODUCTION

As bone is continuously remodeled, bone mass of vertebrates is constantly changing and determined by the balance of bone resorption and bone formation. In adolescence, bone formation predominates until the peak bone mass is reached. From then on, bone mass continuously declines, with certain events such as the hormonal changes in female menopause, accelerating the process (Bernstein, American Academy of Orthopaedic Surgeons, American Academy of Family Physicians, & American Academy of Pediatrics, 2003). Reduced bone mass is associated with reduced stability of bones in humans, leading to pathological conditions such as fragility fractures, which are a significant health problem, especially as life expectancy is constantly increasing (Compston et al., 2019; Kanis, 2002). Bone loss can be caused by reduced bone formation as well as increased bone resorption, and many factors contributing to bone loss have been identified (Compston et al., 2019; Laine et al., 2013).

miR‐146a is a predominantly anti‐inflammatory microRNA that has been shown to be important in various aspects of inflammation and immunity (Magilnick et al., 2017; Taganov et al., 2006). It has primarily been reported as an anti‐inflammatory miRNA, as deficiency in miR‐146a results in increased inflammation in various instances (Boldin et al., 2011; Lu et al., 2010; Saferding et al., 2017). This miRNA has also been shown to be associated with aging, as it is one of the microRNAs that was demonstrated to increase with age (Jiang et al., 2012), while it is reduced by hormone replacement therapy in postmenopausal women (Kangas et al., 2014).

Several miRNAs, among them miR‐146a, have been implicated in regulating the biology of bone, both osteoblasts/osteocytes and osteoclasts (Bluml et al., 2011; Hu et al., 2017; Krzeszinski et al., 2014; Liu et al., 2019; Weilner et al., 2016; Zeng et al., 2017). We have previously demonstrated that the loss of miR‐146a increases the severity of inflammatory arthritis by regulating fibroblast pathogenicity, especially their ability to induced bone‐resorbing osteoclasts (Saferding et al., 2017). However, the role of miR‐146a in bone homeostasis is not known. Here, we describe the microRNA‐146a as a critical factor, which regulates bone loss during aging. Mice deficient in this miRNA constantly accrue bone over time, leading to a high bone mass phenotype. Loss of miR‐146a also protects from ovariectomy‐induced bone loss. In addition, in humans we find increased levels of miR‐146a in patients suffering fragility fractures compared with those who had no fractures, suggesting a crucial role of miR‐146a in bone homeostasis.

## 2 RESULTS

### 2.1 miR‐146a‐deficient mice continuously accumulate bone during aging

**Figure 1:**
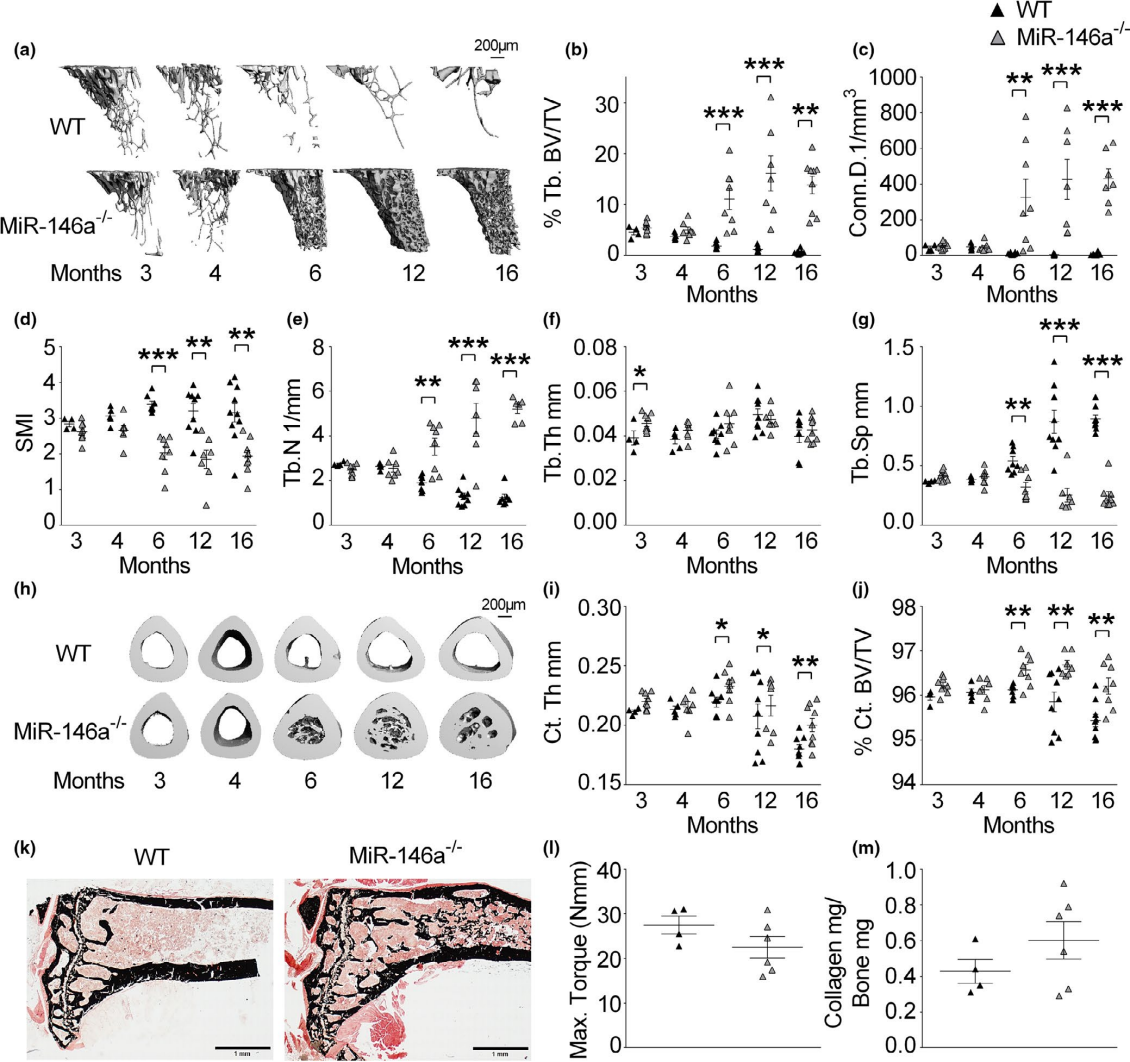
miR‐146a‐deficient mice continuously accumulate bone during aging. (a) Representative µCT images of trabecular bones from tibial WT and miR‐146a^−/−^ animals aged 3–16 months (bar 200 µm). (b–g) Three‐dimensional reconstruction and quantitation of the indicated parameters of trabecular bone from proximal tibias of WT and miR‐146a‐deficient animals aged 3–16 months, using µCT (*n* ≥ 4). (h) Representative images of cortical bone from WT and miR‐146a^−/−^ animals aged 3–16 months (bar 200 µm). (i, j) Bone morphometric analysis of the indicated parameters of cortical bone at the diaphysis of the tibia, close to the site of the intersecting fibula using µCT (*n* ≥ 4). (k) Von Kossa staining of histological sections, obtained from the proximal region of tibias from 6‐month‐old WT and miR‐146a^−/−^ mice, and representative images are shown (bars 1 mm, magnification ×2.5). (l and m) Maximal torque at the femoral shaft assessed by three‐point bending and collagen mg/bone mg analyzed in femoral bone in 6‐month‐old WT and miR‐146a^−/−^ animals (*n* ≥ 4). Tb.BV/TV, trabecular bone volume per tissue volume; Conn.D, connectivity density; SMI, structural model index; Tb.N, trabecular number; Tb.Th, trabecular thickness; Tb.Sp, trabecular separation; Ct.Th, cortical thickness; Ct.BV/TV, cortical bone volume per tissue volume; all data shown were obtained from female animals. *p < 0.05; **p < 0.01; ***p < 0.001. Results are shown as mean ± *SEM*

To investigate the impact of miR‐146a on bone biology, we used miR‐146a full knockout animals and assessed trabecular and cortical bone parameters in tibial bones of animals aged 3–16 months. Micro‐computed tomographic (μCT) analysis showed regular development of the skeleton and bone volume until 4 months of age until reaching the peak bone mass, which is at 3‐4 months in C57BL/6 mice (Beamer et al., 1996). While WT mice started to lose bone mass from then on, as expected, miR‐146a‐deficient mice continued to accrue bone until 12 months of age, with bone mass remaining stable until 16 months of age (Figure 1a–g). miR‐146a‐deficient mice displayed elevated trabecular bone volume per tissue volume (BV/TV), trabecular number (Tb.N), connectivity density (Conn.D), and reduced trabecular separation (Tb.Sp) compared with WT mice starting at 6 months of age throughout 16 months, while trabecular thickness (Tb.Th) was not changed between these two groups. Moreover, the structural model index (SMI) was significantly changed in miR‐146a‐deficient animals, indicating a rather plate‐like than rod‐like geometry of trabecular bone in the absence of miR‐146a. In addition to trabecular measures of bone volume, cortical BV/TV and cortical thickness (Ct.Th) also increased and cortical porosity decreased significantly with age in miR‐146a^−/−^ mice compared with control animals (Figure 1h–j). Similar to the observations in trabecular bone, cortical bone parameters in both groups such as cortical thickness (Ct.Th), cortical BV/TV, and cortical porosity were not different until 4 months of age. However, starting from 6 months of age, cortical thickness and BV/TV increased, and cortical porosity decreased significantly in miR‐146a‐deficient mice compared with WT mice (Figure 1i–k and Figure S1A). Bone mineral density (BMD) solely increased at 16 months of age in knockout animals (Figure S1B). Of note, aged miR‐146a‐deficient animals show massive trabecular bone growth reaching into the diaphyseal shaft of the tibia, which is normally devoid of trabecular bone in WT animals, indicating a strongly deregulated bone growth in miR‐146a^−/−^ animals (Figure 1h). Interestingly, this bone phenotype was predominantly found in female mice, whereas male mice showed only a slight increase in their bone mass at 12 months of age (Figure S2). To analyze the mechanical properties of bone from miR‐146a‐deficient animals, we performed 3‐point bending assays. Bone strength of mice aged 6 months was not altered compared with control animals, and we detected an equal amount of collagen in bone of 6‐month‐old WT and miR‐146a^−/−^ animals (Figure 1l,m).

**Figure 2:**
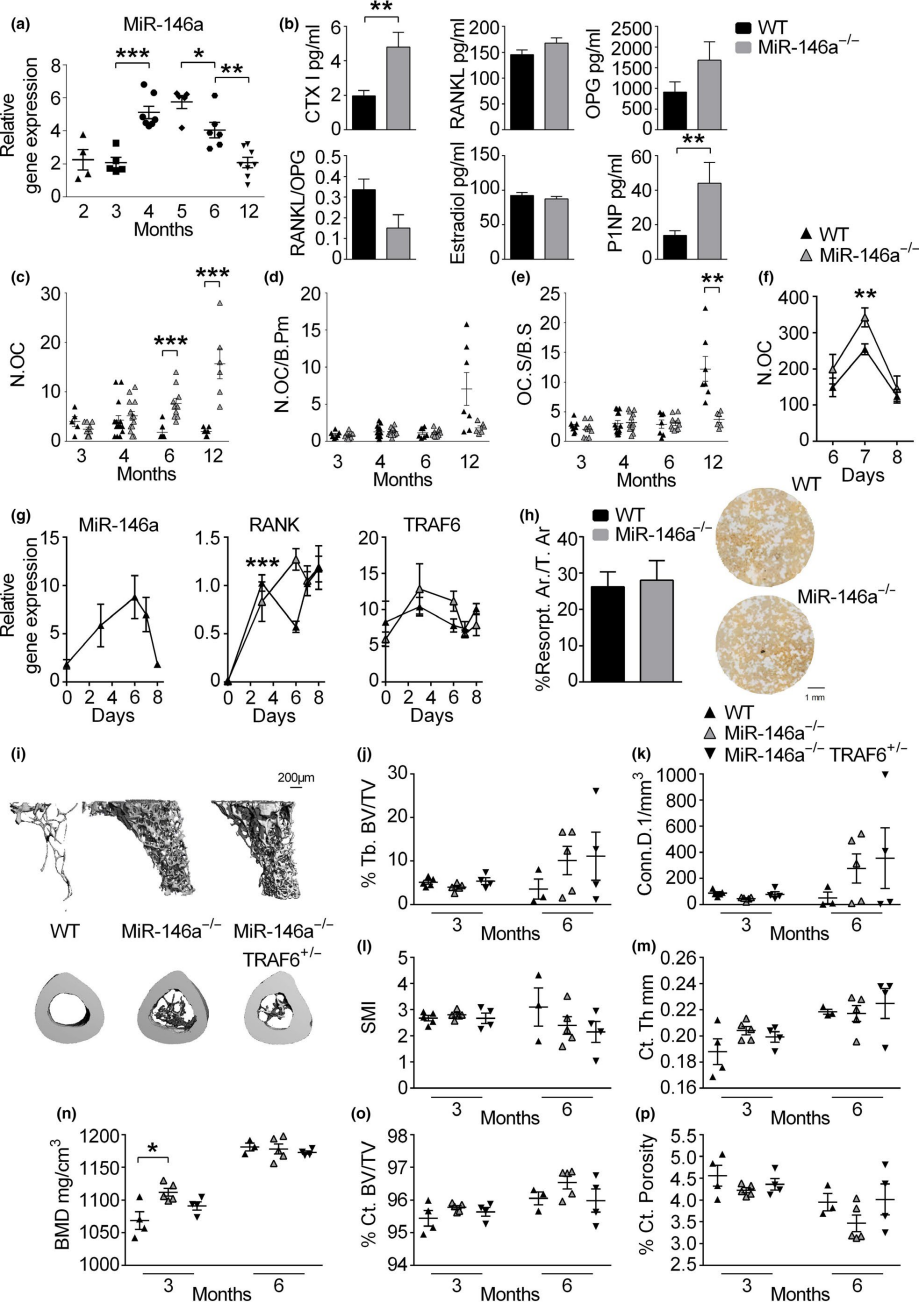
Osteoclast activity is not responsible for high bone mass in miR‐146a‐deficient animals. (a) Expression level of miR‐146a in femoral bone of WT animals aged from 2 to 12 months using quantitative real‐time PCR (qPCR) analysis (*n* ≥ 4). (b) Levels of CTX, RANKL, OPG, estradiol, P1NP, and RANKL/OPG ratio were measured in sera of 6‐month‐old WT and miR‐146a^−/−^ mice using ELISA (*n* ≥ 4). (c–e) Histomorphometric analysis of tartrate‐resistant acid phosphatase (TRAP)‐stained tibial sections, N.OC, N.OC/B.Pm, and OC.S/BS of WT and miR‐146a^−/−^ mice aged 3 to 12 months were assessed (*n* ≥ 5). (f) Bone marrow‐derived osteoclasts were generated and stained for TRAP 6, 7, and 8 days after bone marrow isolation, RANKL was added on days 3 and 6 (*n* ≥ 3). (g) Expression level of miR‐146a, RANK, and TRAF6 in bone marrow‐derived osteoclasts from 3‐month‐old WT and miR‐146a^−/−^ animals was analyzed using qPCR. Bone marrow was isolated on day 0, and cells were cultured with MCSF over 8 days and stimulated with RANKL on days 3 and 6 (*n* = 4). (h) Quantification of osteoclast resorption capacity (left) of WT and miR‐146a‐deficient animals. Representative images of in vitro bone resorption assays are shown (right, bar 1 mm) (*n* = 8). (i) Representative µCT images of trabecular and cortical bone of 6‐month‐old WT, miR‐146a^−/−^, and miR‐146a^−/−^ TRAF6^+/−^ animals are shown (bar 200 µm). (j–p) Bone morphometric analysis of trabecular bone from proximal tibias and cortical bone at the diaphysis of the tibia (close to the site of the intersecting fibula) of 3‐ and 6‐month‐old WT, miR‐146a^−/−^, and miR‐146a^−/−^ TRAF6^+/−^ mice (*n* ≥ 3). N.OC, numbers of osteoclasts; N.OC/B.Pm, numbers of osteoclasts per bone perimeter; OC.S/BS, osteoclast surface per bone surface; CTX, C‐terminal telopeptide of type I collagen; RANKL, receptor activator of NF‐κB ligand; OPG, osteoprotegerin; P1NP, procollagen type 1 N‐terminal propeptide; Tb.BV/TV, trabecular bone volume per tissue volume; Conn.D, connectivity density; SMI, structural model index; Ct.Th, cortical thickness; BMD, bone mineral density; Ct.BV/TV, cortical bone volume per tissue volume; Ct. Porosity; cortical porosity; all analyses were performed in female animals. *p < 0.05; **p < 0.01; ***p < 0.001. Results are shown as mean ± *SEM*

As the increase in bone mass was developing with age, we investigated whether miR‐146a levels were differentially regulated over time in bone of WT animals. Young and early adult mice at the age of 2 and 3 months showed no difference in the expression level of miR‐146a in bone tissue (Figure 2a). However, starting at 4 months of age, expression levels of this microRNA increased significantly, reaching a maximum at 5 months of age. Even though it declined from 5 to 6 months of age, its expression was still increased at this point, finally dropping in tissue samples of 12‐month‐old animals (Figure 2a). These data demonstrate elevated miR‐146a expression in bone around peak bone mass, and the course of miR‐146a expression correlates with the switch from bone accrual to bone loss, supporting the notion of a regulatory function of miR‐146a in bone during aging.

Following, we examined known regulators of bone turnover in sera of WT and miR‐146a^−/−^ animals. miR‐146a^−/−^ animals showed elevated levels of the bone resorption marker C‐terminal telopeptide of type I collagen (CTXI) at 6, 12, and 18 months of age (Figure 2b and Figure S3) as well as the bone formation marker P1NP (Figure 2b). Since the bone phenotype was more profound in female animals, we assessed estrogen levels, which were not different between WT and knockout animals. Moreover, we did detect a trend toward a reduced RANKL/OPG ratio in miR‐146a‐deficient mice compared with WT mice which was primarily driven by increased levels of OPG in miR‐146a^−/−^ animals compared with WT animals (Figure 2b).

Analysis of marker genes of osteoblasts including Runt‐related transcription factor 2 (Runx2), osteocalcin (OC), alkaline phosphatase (ALP), osterix (OSX), osteopontin (OPN), and osteoclasts, including tartrate‐resistant acid phosphatase (TRAP), cathepsin K (CTSK), and receptor activator of NF‐κB (RANK) in femoral bones of WT and miR‐146a^−/−^ animals, did not show any difference at 3 and 6 months of age. However, RUNX2, OSX, OPN, BMP2, and RANKL and TRAP, CTSK, and RANK increased significantly at 12 months of age in miR‐146a‐deficient bones (Figure S4A,B). Taken together, these data demonstrate that both osteoblast and osteoclast‐associated genes are deregulated in miR‐146a^−/−^ mice during aging.

Therefore, we investigated static histomorphometry of WT and miR‐146a‐deficient mice. Histological assessment of bone microarchitecture paralleled our data obtained in µCT analysis (Figure S5A–D). Although overall numbers of osteoclasts (N.OC) were elevated in 6‐ and 12‐month‐old miR‐146a^−/−^ animals compared with WT animals, we could not detect a difference in osteoclast number per bone perimeter (N.OC/B.Pm) or osteoclast surface per bone surface (OC.S/BS) at all earlier time points up to 6 months. However, 12‐month‐old mice deficient in miR‐146a displayed reduced OC.S/BS and N.OC/B.Pm (Figure 2c–e).

To explore whether osteoclast differentiation and function are changed in mice lacking miR‐146a, we performed analysis of WT and miR‐146a^−/−^ osteoclasts. Already published investigation of in vitro differentiated osteoclasts upon stimulation with RANKL had revealed no difference in bone marrow‐derived osteoclast numbers between WT and miR‐146a‐deficient animals (Saferding et al., 2017). We extended these analyses to determine the kinetics of OC differentiation. We detected a slightly increased capacity of miR‐146a‐deficient bone marrow cells to differentiate into OCs only at one time point, with similar kinetics and survival as WT OCs (Figure 2f). We further examined expression levels of miR‐146a during osteoclastogenesis, which significantly increased initially after stimulation with RANKL and dropped back to baseline levels in mature OCs (Figure 2g). The tumor necrosis factor receptor‐associated factor 6 (TRAF6) had been shown to be essential in RANKL‐induced osteoclast generation, as TRAF6 is pivotal in RANK‐mediated signal transduction and also to be a direct target of miR‐146a (Lomaga et al., 1999; Taganov et al., 2006). However, gene expression analysis of RANK and TRAF6 during in vitro differentiating osteoclasts did not show any difference between WT and miR‐146a knockout animals, suggesting no regulation of TRAF6 levels by miR‐146a during in vitro differentiating OCs (Figure 2g). Moreover, the function of in vitro differentiated OCs was not altered, as bone resorption capacity was not different between the two groups (Figure 2h). In addition, TRAF6 expression levels in bone measured by qPCR were not different at all time points analyzed, as were mRNA levels of SMAD4, another prominent experimentally confirmed target of miR‐146a (Figure S4C).

To investigate whether deregulation of TRAF6 in vivo was responsible for the bone phenotype we observed in miR‐146a‐deficient mice, we analyzed WT, miR‐146a^−/−^, and miR‐146a^−/−^/TRAF6^+/−^ animals. These miR‐146a^−/−^/TRAF6^+/−^ mice were shown to express TRAF6 levels similar to WT animals, leading to the resolution of some important phenotypic observations in miR‐146a‐deficient mice, such as aberrant myeloproliferation, splenomegaly, and excessive inflammatory responses, but not others (Magilnick et al., 2017). However, there were no significant differences in the BV/TV or other parameters analyzed in µCT between miR‐146a^−/−^ and miR‐146a^−/−^/TRAF6^+/−^ mice, neither in trabecular nor in cortical bone (Figure 2i‐p and Figure S6A‐C), demonstrating that deregulation of TRAF6 in miR‐146a‐deficient mice is not responsible for the observed bone phenotype. Taken together, these data suggest that bone accrual in miR‐146a‐deficient mice starting at 6 months of age was not due to decreased activity or numbers of bone‐resorbing osteoclasts.

### 2.2 Increased activity of osteoblasts in vivo in aged miR‐146a‐deficient mice

**Figure 3:**
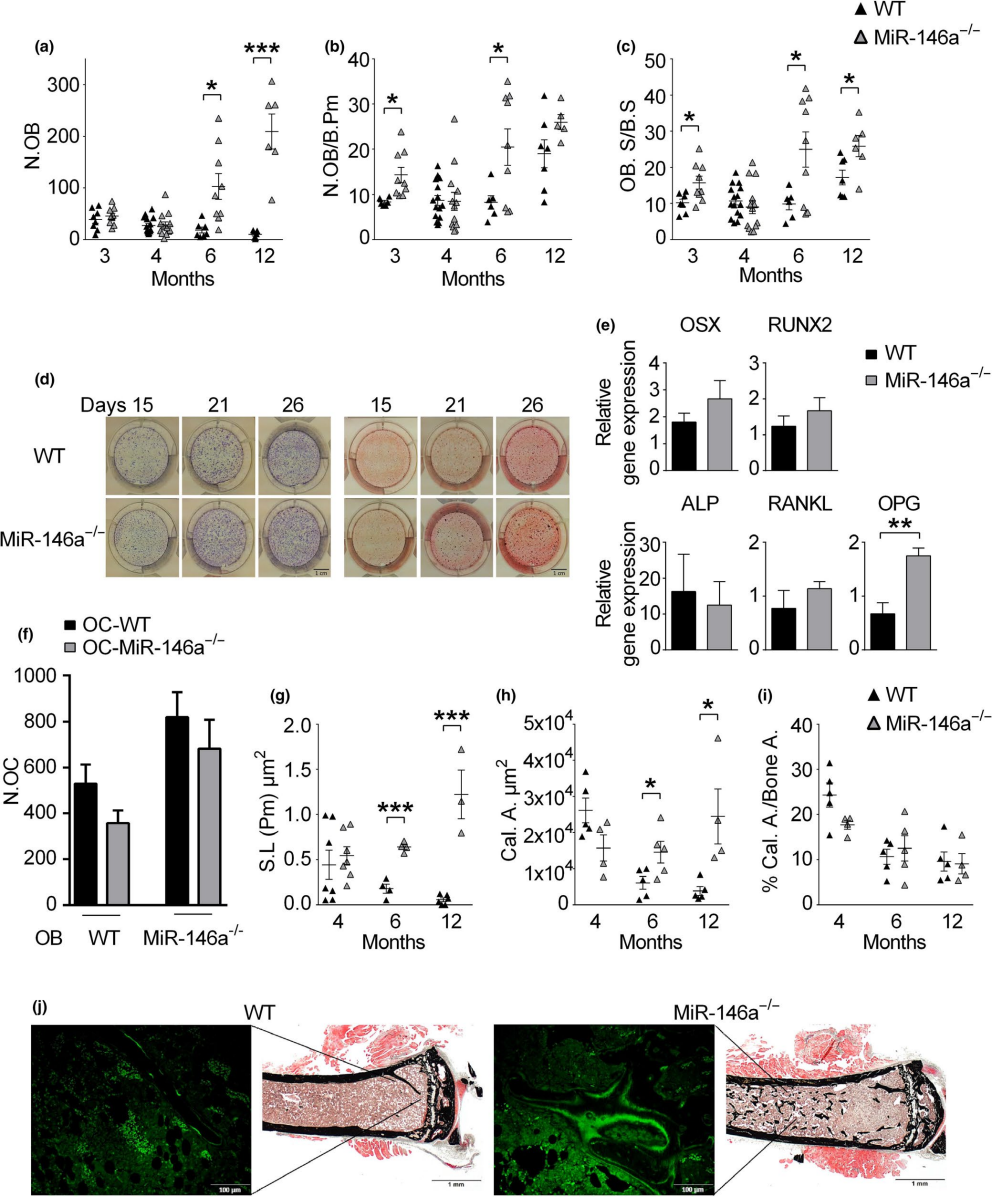
Increased activity of osteoblasts in vivo in aged miR‐146a‐deficient mice. (a–c) Histomorphometric analysis of N.OB, N.OB/B.Pm, and OB.S/BS of WT and miR‐146a^−/−^ mice aged 3–12 months of TRAP‐stained tibial sections (*n* ≥ 5). (d) Representative images of alkaline phosphatase (left) and Alizarin Red (right) staining of WT and miR‐146a^−/−^ osteoblasts after 15, 21, and 26 days of osteogenic differentiation (bars 1 cm, *n* = 4). (e) Gene expression of OSX, RUNX2, ALP, RANKL, and OPG was measured in osteoblasts of WT and miR‐146a^−/−^ mice after 21 days of osteogenic differentiation using qPCR (*n* ≥ 3). (f) Numbers of osteoclasts were analyzed in co‐cultures of either WT or miR‐146a^−/−^ osteoblasts cultured with WT or miR‐146a‐deficient bone marrow cells, stimulated with vitamin D3 and dexamethasone for 7 days (*n* = 3). (g–i) WT and miR‐146a^−/−^ animals aged 4–12 months were labeled with calcein at two time points (6 and 1 days before sacrifice), and histological sections of the proximal tibia were analyzed for S.L., Cal.A, and Cal.A/Bone A. (n ≥ 4). (j) Representative images of on Kossa (left, bars 1 mm magnification ×2,5) and calcein labeled (right, bars 100 µm magnification ×20) tibial sections of WT and miR‐146a^−/−^ animals 6 months of age. N.OB, number of osteoblasts; N.OB/B.Pm, number of osteoblasts per bone perimeter; OB.S/BS, osteoblast surface per bone surface; OSX, osterix; ALP; alkaline phosphatase; RANKL, receptor activator of NF‐κB; OPG, osteoprotegerin; S.L, single label; Cal.A, calcein area; Cal.A/Bone A, calcein area per bone area; all data shown were generated from female animals. *p < 0.05; **p < 0.01; ***p < 0.001. Results are shown as mean ± *SEM*

Analyzing osteoblast‐related parameters, we detected significantly increased numbers of osteoblasts per bone perimeter (N.OB/B.Pm) and osteoblast surface per bone surface (OB.S/BS) in miR‐146a‐deficient mice, at 3, 6, and 12 months of age compared with their WT counterparts (Figure 3a–c). However, investigation of in vitro differentiation of calvarial osteoblasts did not reveal any difference in ALP staining, nor changes in the ability of OBs to induce bone nodule formation between WT and miR‐146a^−/−^ animals, suggesting no major intrinsic defects in osteoblast differentiation in vitro (Figure 3d). In line with this observation, osteoblast‐related marker genes such as Runx2, osterix, or ALP did not show any significant differences between in vitro differentiated OBs of the two groups, only OPG was significantly increased in miR‐146a‐deficient OBs compared with WT OBs (Figure 3e). To investigate whether OBs lacking miR‐146a exhibit differences in their capacity to induce osteoclastogenic differentiation of bone marrow cells, we performed co‐culture assays. Calvaria‐derived mesenchymal stem cells were cultured with bone marrow cells as myeloid responders, stimulated with vitamin D3 and dexamethasone. WT osteoblasts induced robust generation of osteoclasts, regardless of the genotype of the myeloid responder cells. Osteoblasts lacking miR‐146a were similarly capable of stimulating osteoclastogenesis, again regardless of the genotype of the responder cells (Figure 3f).

To examine dynamic bone formation, we performed calcein labeling in WT and miR‐146a‐deficient mice. While in WT animals single‐ or double‐layer labeled bone surfaces were easily discernible, irregular incorporation of calcein occurred in miR‐146a‐deficient animals, which precluded standard analysis of bone formation and mineral apposition rate. Therefore, we quantified total calcein incorporation in WT and miR‐146a‐deficient mice. We detected significantly increased single‐layer calcein incorporation (Figure 3g) as well as an elevated total area of calcein incorporation (Figure 3h–j) in miR‐146a‐deficient mice compared with WT mice starting at 6 months of age, indicating enhanced total activity of osteoblasts in miR‐146a‐deficient mice.

### 2.3 Increased Wnt expression and signaling in aged miR‐146a‐deficient mice

**Figure 4:**
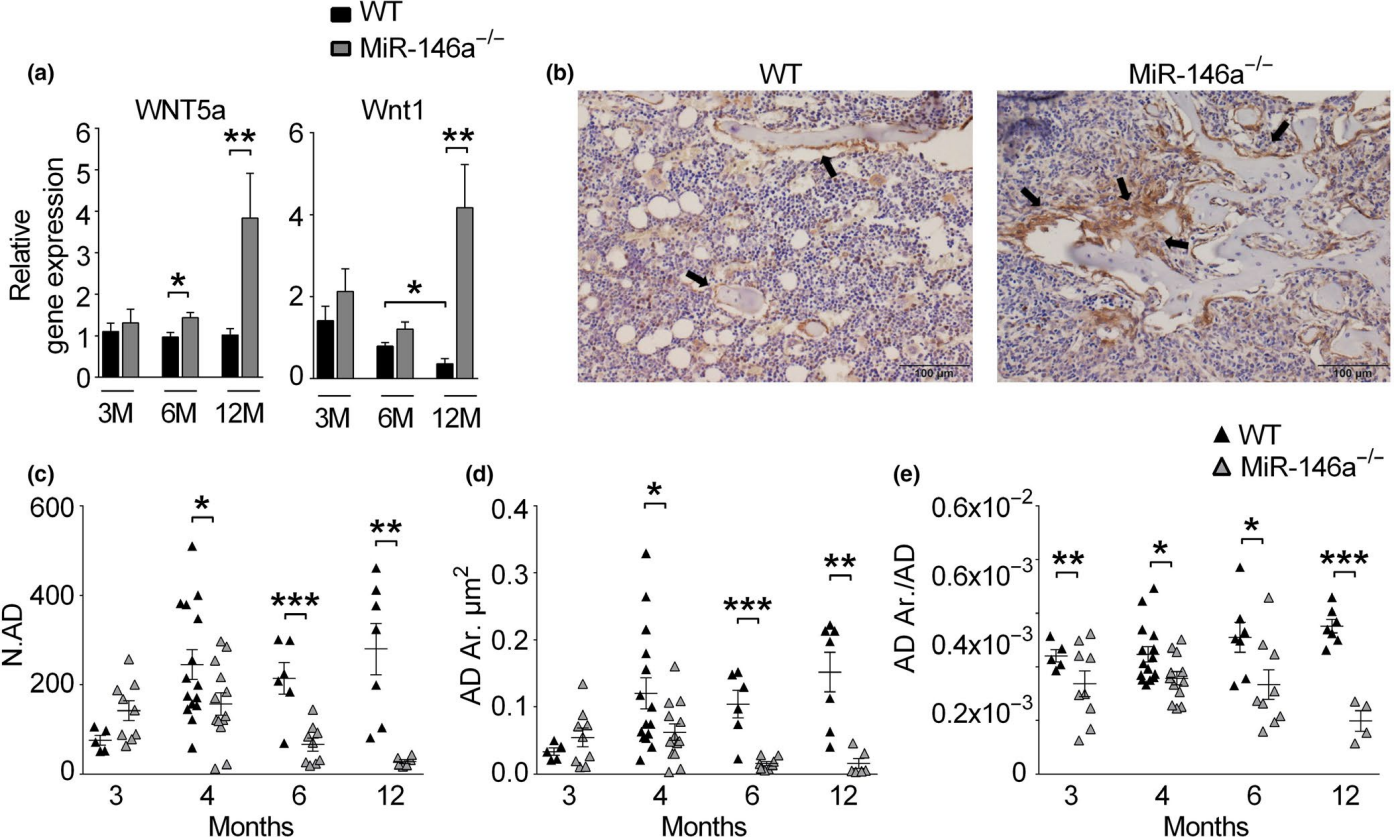
Loss of miR‐146a increases Wnt signaling and prevents bone marrow adiposity with age. (a) Gene expression analysis of Wnt5a and Wnt1 in femoral bones of 3‐ to 12‐month‐old WT and miR‐146a^−/−^ mice using qPCR (*n* ≥ 6). (b) Representative pictures of immunohistochemically stained tibial sections of 6‐month‐old WT and miR‐146a‐deficient animals using β‐catenin antibody (bars 100 µm, magnification ×20). Black arrows indicate positively stained osteoblasts at sites of bone formation. (c–e) Histomorphometric analysis of N.AD, AD. Ar, and AD. Ar/AD from histological sections of 3–12 months aged WT and miR‐146a^−/−^ animals (*n* ≥ 4). N.AD, number of adipocytes; AD.Ar, adipocyte area; AD.Ar/AD, adipocyte area per adipocyte; all analyses shown were obtained from female animals. *p < 0.05; **p < 0.01; ***p < 0.001. Results are shown as mean ± *SEM*

Previous reports have demonstrated that miR‐146a directly targets members of the Wnt family of proteins such as Wnt1, Wnt3, and Wnt5a (Du et al., 2015; Sun et al., 2017). Wnt5a and Wnt1 were of particular interest, as mice deficient in these proteins have low bone mass due to reduced activity of osteoblast (Joeng et al., 2014, 2017; Maeda et al., 2012). Analyzing RNA expression in bone, we detected similar levels of Wnt5a in bone tissue of mice aged 3 months. In contrast, Wnt5a levels in bone were significantly increased in mice lacking miR‐146a aged 6 months or older. In addition, while there was a trend toward increased expression of Wnt1 at 3 and 6 months of age, 12‐month‐old mice displayed significantly increased expression of Wnt1 (Figure 4a). These data demonstrate that Wnt family members, especially Wnt5a, are increased in aged but not in young miR‐146a‐deficient mice. Wnt signaling, induced both by Wnt5a and by Wnt1, leads to increased stability of the transcription factor β‐catenin and therefore increased the presence of this protein (Baron & Kneissel, 2013; Cadigan & Peifer, 2009). In line with augmented Wnt signaling, we detected markedly elevated levels of β‐Catenin in bone tissue, and specifically in osteoblasts of aged miR‐146a‐deficient mice compared with WT mice (Figure 4b arrows), demonstrating increased Wnt signaling in bone of miR‐146a‐deficient mice compared with WT mice.

Wnt5a in particular was shown to be involved regulating differentiation of mesenchymal stem cells, where it drives osteogenesis at the expense of adipogenesis (Bilkovski et al., 2010). Since we had observed increased numbers of osteoblasts in aged miR‐146a‐deficient mice, we next investigated bone marrow adiposity in those mice. Indeed, we found significantly increased numbers of adipocytes in WT mice compared with miR‐146a‐deficient mice, suggesting increased osteoblast generation accompanied by decreased adipocyte generation and bone marrow adiposity during aging in miR‐146a‐deficient mice compared with WT mice (Figure 4c‐e).

### 2.4 miR‐146a deficiency protects from ovariectomy‐induced bone loss

**Figure 5:**
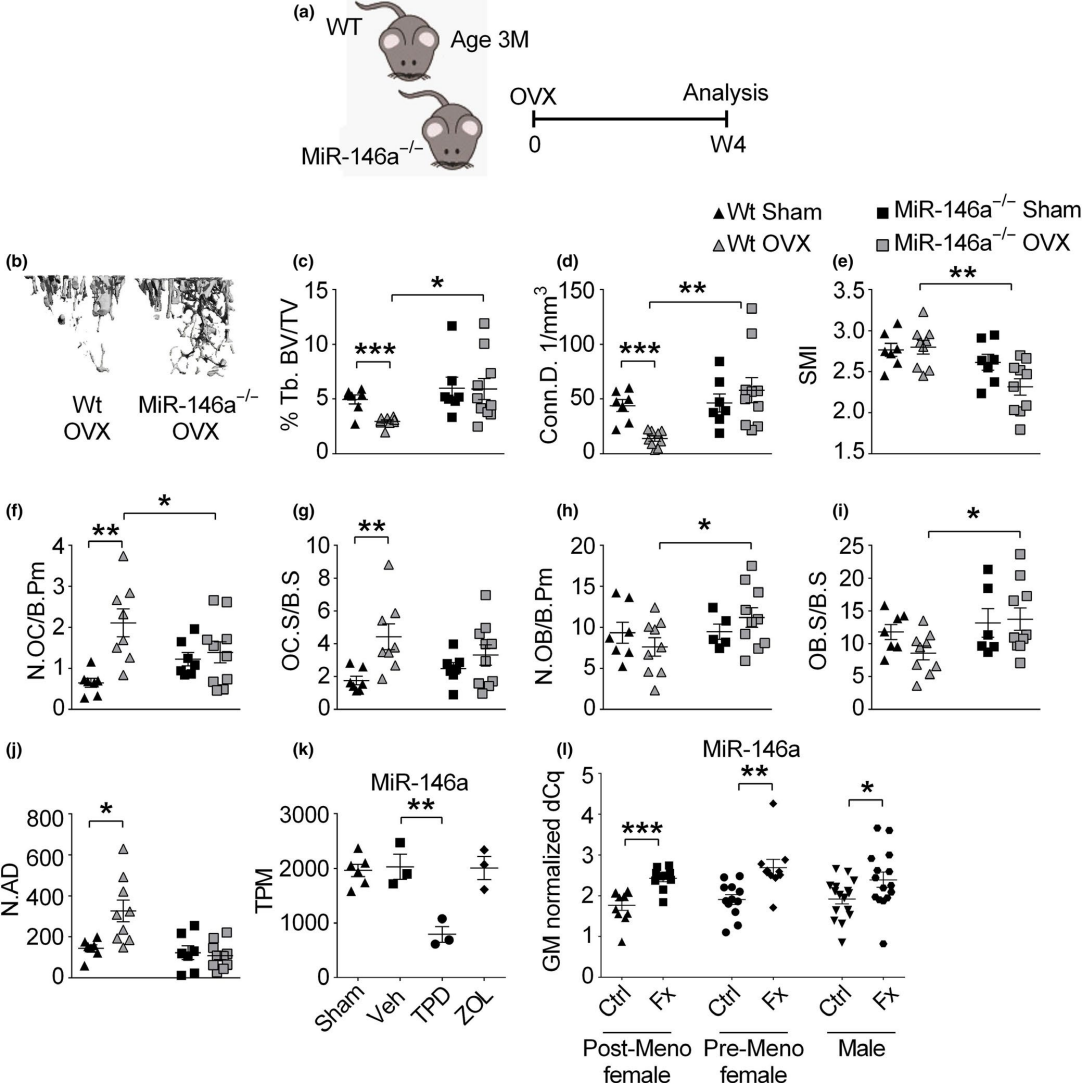
Loss of miR‐146a protects from ovariectomy‐induced bone loss and is increased in patients with fragility fractures. (a) Schematic illustration of the ovariectomy‐induced bone loss experiment performed in 3‐month‐old WT and miR‐146a^−/−^ animals. (b) Representative µCT images of tibial trabecular bones from ovariectomized WT and miR‐146a‐deficient mice (bar 200 µm). (c–e) Three‐dimensional reconstruction and quantitation of Tb.BV/TV, Conn.D., and SMI of the trabecular tibial bone from sham as well as ovariectomized WT and miR‐146a^−/−^ mice using µCT (*n* ≥ 7). (f–j) Histomorphometric analysis of N.OC/B.Pm, OC.S/BS, N.OB/B.Pm, OB.S/BS, N.OB, and N.AD from TRAP‐stained sections of tibial bone (*n* ≥ 5). (k) miR‐146a levels were analyzed in sera of 6‐month‐old sham‐operated or ovariectomized rats treated with either vehicle, TPD, or ZOL starting 8 weeks after ovariectomy for 12 weeks, using qPCR (*n* ≥ 3). (l) Serum levels of miR‐146a were measured in female postmenopausal and pre‐menopausal and in male patients, control or low traumatic, using qPCR (*n* ≥ 10). Tb.BV/TV, trabecular bone volume per tissue volume; Conn.D., connectivity density; SMI, structural model index; N.OC/B.Pm, numbers of osteoclasts per bone perimeter; OC.S/BS, osteoclast surface per bone surface; N.OB/B.Pm, numbers of osteoblasts per bone perimeter; OB.S/BS, osteoblast surface per bone surface; N. Ad, number of adipocytes TPD, teriparatide; ZOL, zoledronate; TPM, total count per million; FX, fracture; GM, global mean (mean Cq value). All analyses shown, except I, were obtained from female animals. *p < 0.05; **p < 0.01; ***p < 0.001. Results are shown as mean ± *SEM*

To evaluate the role of this miRNA in osteoporosis development, we used the murine ovariectomy (OVX)‐induced model of postmenopausal osteoporosis. We performed OVX in 3‐month (12 weeks)‐old WT and knockout animals, and sham‐operated animals were used as controls. In line with our previous analyses, baseline µCT data showed no differences in bone parameters at this stage of aging (Figure 5c). After 4 weeks, ovariectomized WT animals showed robust bone loss compared with sham‐operated mice. In contrast, OVX in miR‐146a‐deficient mice did not result in any detectable bone loss compared with sham‐operated miR‐146a‐deficient mice (Figure 5a–e and Figure S7A–G). Moreover, histological analysis of bone‐resorbing and bone‐forming cells showed elevated numbers and activity of osteoclasts, as determined by increased number of osteoclasts per bone perimeter and osteoclast surface per bone surface, in WT OVX animals compared with sham‐operated mice. In contrast, miR‐146a‐deficient animals showed no change in osteoclast numbers and activity (Figure 5f,g). On top of that, osteoblast numbers per bone perimeter and osteoblast surface per bone surface even increased in OVX knockout animals compared with WT animals (Figure 5h–i), suggesting increased activity of osteoblasts in miR‐146a‐deficient compared with WT mice after OVX. In addition, while bone marrow adiposity, measured by the number of adipocytes, increased significantly in WT mice after OVX, we detected no change in miR‐146a‐deficient mice after OVX (Figure 5j). Summarized, these data reveal reduced activation of osteoclast and preserved activity of osteoblasts in ovariectomized miR‐146a‐deficient mice, leading to complete protection from OVX‐induced bone loss.

To investigate how current treatment regimens for estrogen‐deficient bone loss influence miR‐146a expression in bone tissue, we reanalyzed data from an OVX rat model in wild‐type animals, using sham‐operated animals as controls. After 8 weeks, OVX animals received either vehicle (Veh), anti‐resorptive treatment using zoledronate (ZOL), or osteoanabolic treatment using teriparatide (TPD), a recombinant parathyroid hormone (PTH) shown to increase bone mass of rats, for 12 weeks (Kocijan et al., 2020). Analyzing microRNA expression in bone, we found that, in line with our hypothesis, treatment with TPD significantly reduced the levels of miR‐146a in bone tissue, correlating with an almost complete protection from OVX‐induced bone loss. In contrast, treatment with bisphosphonates, which target osteoclast‐mediated bone resorption, did not alter levels of miR‐146a in bone. (Figure 5k). These data demonstrate that the treatment of OVX‐induced bone loss with recombinant PTH is associated with suppression of miR‐146a.

### 2.5 Increased expression of miR‐146a in patients with fragility fractures

Finally, in order to investigate whether miR‐146a could also be involved in human disease, we tested the levels of miR‐146a in patients with osteoporosis, reanalyzing an existing dataset (Kocijan et al., 2016). In line with our murine data, women suffering from low trauma fractures had increased circulating serum levels of miR‐146a compared with those who did not (Figure 5l). There was even a significant difference in pre‐menopausal fractures in females compared with controls. In men, miR‐146a was significantly elevated in individuals who suffered a fracture as well. However, the difference was less pronounced in males compared with females, pointing toward a predominant role of miR‐146a in the regulation of bone in female individuals.

## 3 DISCUSSION

In this study, we identify a novel and unexpected role of miR‐146a in bone homeostasis. miR‐146a increases with age and acts as an epigenetic molecular switch terminating bone accrual, leading to bone loss during aging by downregulating bone anabolic Wnt signaling. Conversely, loss of miR‐146a leads to continuous bone accrual during aging as a result of unrestricted bone anabolism and intact Wnt signaling. Therefore, miR‐146a crucially controls the temporal facet of bone homeostasis.

miR‐146a has been primarily identified as an anti‐inflammatory miRNA, as it has been shown to inhibit many aspects of inflammation, hematopoietic stem cell biology, and cancer (Boldin et al., 2011; Lu et al., 2010; Magilnick et al., 2017; Nakasa et al., 2008; Zhao et al., 2011, 2013). However, miR‐146a has also been implicated in immune dysfunction during aging, as it has been shown to accumulate in macrophages and dendritic cells of aged mice, leading to dysfunction of these cells (Jiang et al., 2012; Olivieri et al., 2013). Myeloproliferation, systemic inflammation, and myelofibrosis are important phenotypes that develop in miR‐146a‐deficient mice with age (Boldin et al., 2011; Magilnick et al., 2017). While TRAF6 has been shown to mediate the systemic inflammatory and myeloproliferative phenotype, TRAF6 haploinsufficiency was not able to rescue the high bone mass phenotype in miR‐146a‐deficient mice. While we were not able to definitely rule out an influence of other phenotypes on the development of high bone mass, we did observe a high bone mass at 6 months of age, where there were no signs of the myeloproliferative phenotype yet (Figure S8). In addition, alterations in bone marrow cellularity at 6 months of age were similar between male and female miR‐146a‐deficient mice (Figure S8 and data not shown), yet only female miR‐146a‐deficient develop a high bone mass phenotype. Conditional miR‐146a‐deficient animals will further help to clarify the mechanisms of the development of high bone mass in the miR‐146a‐deficient mice.

miR‐146a was found to suppress the osteogenesis of adipose‐derived mesenchymal stem cells by targeting SMAD4 (Xie et al., 2017). Since SMAD4 was not differentially expressed in our analysis of miR‐146a‐deficient bone, in neither young nor aged animals it does not seem to be responsible for the elevated bone growth in knock out animals.

Of note, we did not detect major differences of in vitro generated osteoclasts or osteoblasts, which contrast some published reports (Kuang et al., 2017). However, these data were primarily obtained upon manipulation of wt osteoblasts with antagomirs or miR‐146a agonists, while our analyses were done ex vivo with osteoblasts of wt and miR‐146a‐deficient mice, possibly explaining the difference outcomes.

The role of Wnt proteins and their receptors in bone biology is well‐established (Baron & Kneissel, 2013; Cadigan & Peifer, 2009). The role of Wnt5a is of particular interest, as it has been implicated in osteoclast activity as well as osteoblast differentiation and activity. The net effect in full Wnt5a knockout mice is reduced bone mass, accompanied by severely reduced activity of osteoblasts (Maeda et al., 2012). Loss of Wnt5a specifically in osteoclasts leads to reduced bone mass due to decreased bone formation, whereas deletion of its receptor Ror2 in OCs leads to increased bone mass, suggesting that Wnt5a activates both osteoblasts and osteoclasts, with a predominant effect on osteoblast function (Maeda et al., 2012; Roberts et al., 2020). Mutations in Wnt1 have been shown to be responsible for osteoporosis and osteogenesis imperfecta, and transgenic overexpression of Wnt1 has been shown to markedly increase bone mass in mice (Laine et al., 2013; Luther et al., 2018). In this study, we found deregulation of Wnt5a as well as Wnt1 accompanying the increased osteoblast activity in miR‐146a‐deficient mice. Importantly, young mice show similar levels of Wnt5a and Wnt1 in WT and miR‐146a‐deficient mice, demonstrating that Wnt5a is temporally regulated, as it is the case with miR‐146a. The fact that timing of Wnt5a expression is crucial for some of its effects has also been demonstrated in embryogenesis, with the help of tamoxifen‐inducible Wnt5a expression in mice (van Amerongen et al., 2012). Wnt proteins have been shown to decline with age in bone (Bakker et al., 2012; Rauner et al., 2008). We also find a decline in Wnt1 in our analyses in WT mice, but not in miR‐146a‐deficient mice. In addition, classical but also sometimes non‐classical Wnt signaling leads to stabilization of β‐catenin (Okamoto et al., 2014). Increased stabilization of β‐catenin in osteoblasts has been shown to increase bone mass, whereas deletion leads to reduced bone mass (Glass et al., 2005). In our experiments, aged miR‐146a‐deficient mice exhibit markedly increased presence of β‐catenin in bone, as a result of increased Wnt signaling. The effects of miR‐146a deficiency are unique, as this miRNA is controlling the temporal aspect of bone turnover. This contrasts with mice deficient in sclerostin, an endogenous inhibitor of Wnt signaling, leading to a high bone mass phenotype already at a younger age (Li et al., 2008). Interestingly, miR‐146 is increased in senescent cells (Deng et al., 2017), whose elimination prevents bone loss with aging (Farr et al., 2017).

Wnt signaling has also been implicated in regulating the fate of mesenchymal stem cells to differentiate into either the osteoblastic or the adipogenic lineage, favoring the former over the latter (Bilkovski et al., 2010; Song et al., 2012). In WT mice, bone marrow adiposity continuously increases with age. In line with increased activity of Wnt proteins, we detected even decreasing bone marrow adiposity and increasing numbers of osteoblasts over time in miR‐146a‐deficient mice. In addition, miR‐146a‐deficient mice are, in accordance with a recent report (Zhao et al., 2019), protected from OVX‐induced bone loss. Interestingly, also during ovariectomy, we detected increased osteoblast and reduced generation of adipocytes in miR‐146a‐deficient mice. As bone marrow adiposity has been shown to increase with age in humans and has been linked with osteoporosis in the elderly as well (Kim et al., 2012), we propose that miR‐146a favors adipocyte generation at the expense of osteoblasts during aging, leading to reduced osteoanabolic capacities as a result of reduced osteoblast differentiation. In addition, miR‐146a deficiency prevented increase in osteoclasts after OVX, suggesting that both mesenchymal and myeloid cells are involved in the protection from OVX‐induced bone loss.

Therefore, the temporal regulation of miR‐146a expression in bone seems to provide the molecular clock, which restricts osteoblast generation by decreasing Wnt5a and Wnt1 expression, tipping the scale toward bone loss during aging in WT mice. This important regulatory function is lost in miR‐146a‐deficient mice, leading to continuous bone accrual during aging. Importantly, the levels of miR‐146a are increased in the circulation of patients suffering from postmenopausal fragility fractures. Therefore, miR‐146a potentially plays an important role in human osteoporosis.

Therefore, our study describes a novel molecular checkpoint controlling age‐related bone loss and could lead to therapeutic targeting of miR‐146a for osteoporosis and age‐related bone loss.

## 4 MATERIALS AND METHODS

### 4.1 Mice

Breeding pairs of miR‐146a^−/−^ and miR‐146a^+/+^ littermate (B6.(FVB)‐MIR146TM1.1BAL/J) mice were provided by Mark Boldin/David Baltimore. Bones from miR‐146a^−/−^ and miR‐146a^−/−^/TRAF6^+/−^ mice were obtained from M. Boldin. Mice used in all experiments were age‐ and sex‐matched. All data were generated from littermates. All animal procedures were approved by the local ethics committee of the Medical University Vienna (BMWFW‐66.009/0228‐WF/V/3b/2017) and were conducted in strict accordance with Austrian law.

### 4.2 Analysis of bone parameters

Histomorphometry and bone formation analysis of mouse tibia from WT and miR‐146a^−/−^ was analyzed as described (Saferding et al., 2017). For von Kossa stainings, tibias were fixed in paraformaldehyde at room temperature for 6 h. Bones were dehydrated using a gradual series of ethanol (70%, 95%, and 100%), infiltrated, and embedded without decalcification in methyl methacrylate, and von Kossa staining was done on tibial sections. For µCT analysis, we used SCANCO Medical µCT 35 to produce images from trabecular and cortical bone and analyzed with SCANCO evaluation software for segmentation, three‐dimensional morphometric analysis, density, and distance parameters.

### 4.3 Immunohistochemistry

For immunohistochemistry, β‐catenin (BD Bioscience) at a dilution of 1:200 was used.

### 4.4 Cell culture

Osteoclast generation was done as previously described (Saferding et al., 2017).

For bone resorption assays, osteoclasts were seeded 600,000 cells/well on osteo‐assay surface plates (Corning) and cultured as previously described (Saferding et al., 2017). After 4 days, osteoclasts were removed using 5% sodium hypochlorite for 5 min and washed two times with water, water was removed, and wells were air‐dried.

Osteoblasts were isolated from calvariae of neonatal WT and miR‐146a^−/−^mice. Calvariae were digested for 10 min in alpha MEM (Gibco) containing 0.1% Collagenase (Sigma) and 0.2% Dispase II (Sigma). Osteogenic precursors were expanded in alpha MEM containing 10% FCS (Gibco) and 1% penicillin /streptomycin. Differentiation of osteoblasts was achieved by adding 0.2 mM l‐ascorbate and 10 mM β‐glycerophosphate (both Sigma). After 15, 21, and 26 days, cells were fixed with 4% formalin, following staining with either Aqua Bidest containing 8% Alizarin Red (Sigma) or staining for alkaline phosphatase using 5‐bromo‐4‐chloro‐3‐indolylphosphate/nitro blue.

### 4.5 miRNA measurement

microRNA was isolated using miRNeasy Mini Kit (Qiagen). miR‐146a expression was measured using TaqMan miRNA assay hsa‐miR‐146a (Applied Biosystems) according to the manufacturer's instruction using the Rotor‐Gene Q PCR cycler (Qiagen). U6snRNA (miRNA assay U6 snRNA Applied Biosystems) was used as internal control. Relative expression of miR‐146a was calculated by the 2ΔΔCT method.

### 4.6 Quantitative real‐time PCR

For mRNA expression analysis, total RNA was isolated from OCs, OBs, and minced femur using RNeasy Mini Kit (Qiagen). CDNA was prepared using the Omniscript RT Kit (Qiagen), followed by SYBR Green‐based quantitative PCR (Roche) using the Light Cycler 480 (Roche). mRNA amounts were normalized relative to glyceraldehyde‐3‐phosphate dehydrogenase (GAPDH) mRNA. Generation of the correct size amplification products was confirmed with agarose gel electrophoresis. The primers for real‐time PCR were as follows: *RANK*: 5′‐CACAGACAAATGCAAACCTTG‐3′ and 5′‐GTCTTCTGGAACCATCTTCCTCC‐3′; *TRAF6*:*5*′‐AAAGCGAGAGATTCTTTCCCTG‐3′ and 5′‐ACTGGGGACAATTCACTAGAGC‐3′; *OSX*: 5′‐GGAGGCACAAAGAAGCCATAC‐3′ and 5′‐TGCAGGAGAGAGGAGTCCATTG‐3′; *RUNX2*: 5′‐TGGCTTGGGTTTCAGGTTAGGG‐3′ and 5′‐TCGGTTTCTTAGGGTCTTGGAGTG‐3′; *ALP*: 5′‐GCTGATCATTCCCACGTTTT‐3′ and 5′‐CTGGGCCTGGTAGTTGTTGT‐3′; *RANKL*: 5′‐TCGTGGAACATTAGCATGGA‐3′ and 5′‐CCTCTCCCAATCTGGTTCAA‐3′; *OPG*: 5′‐TACCTGGAGATCGAATTCTGCTT‐3′ and 5′‐CCATCTGGACATTTTTTGCAAA‐3′; *Wnt5a*: 5′‐CCAACTGGCAGGACTTTCTC‐3′ and 5′‐GCATTCCTTGATGCCTGTCT‐3′; *Wnt1*: 5′‐TTTTGGTCGCCTCTTTGG‐3′ and 5′‐TGCCTCGTTGTTGTGAAGG‐3′; *OC*: 5′‐ACCTTATTGCCCTCCTGCTT‐3′ and 5′‐GCGCTCTGTCTCTCTGACCT‐3′; *OPN*: 5′‐CTCCATCGTCATCATCATCG‐3′ and 5′‐TGCACCCAGATCCTATAGCC‐3′; *TRAP*: 5′‐TCCTGGCTCAAAAAGCAGTT‐3′ and 5′‐ACATAGCCCACACCGTTCTC‐3′; *CatK*: 5′‐TGAGAGTTGTGGACTCTGTGCT‐3′ and 5′‐TTGTGCATCTCAGTGGAAGACT‐3′; *SMAD4*: 5′‐TGGGTCCGTGGGTGGAATAG‐3′ and 5′‐TCTAAAGGCTGTGGGTCCGC‐3′; BMP2: 5′‐CGCTCCACAAACGAGAAAAG‐3′ and 5′‐CAGTCATTCCACCCCACATC‐3′; and GAPDH: 5′‐TGGCATTGTGGAAGGGCTCATGAC‐3′ and 5′‐ATGCCAGTGAGCTTGCCGTTCAGC‐3′. The relative expression of the mRNA of the gene of interest was calculated by the 2ΔΔCT method.

### 4.7 Dynamic labeling of bone

Calcein labeling was performed and analyzed as described (Bluml et al., 2015).

### 4.8 Ovariectomy (OVX)

OVX of 3‐month‐old female WT and miR‐146a^−/−^ animals was performed as described (Scholtysek et al., 2013). Ovaries were removed from OVX animals, skin of sham‐operated female animals was incised, and ovaries were left intact. The histological analysis of surgically removed ovaries was performed to verify OVX. After 4 weeks of recovery and to allow the onset of osteoporosis, all mice were sacrificed. Bone loss was evaluated in the tibiae, of these mice, histologically and using μCT image analysis.

### 4.9 Three‐point bending and mechanical stability

Mechanical properties of femur from WT and miR‐146a^−/−^ (6‐month‐old animals) were tested as described (Stange et al., 2013).

### 4.10 ELISA

Serum concentration of RANKL (R&D Systems), CTXI (BIOZOL/Biomedica), OPG (AbFrontier), P1NP (Immunodiagnostic Systems (IDS)), and estradiol (Calbiotech) was assessed according to the manufacturer's instructions.

### 4.11 Flow cytometry

Cells were stained for CD11b (PerCP‐Cy™5.5‐conj., clone M1/70) BD PharMingen™. For flow cytometric analyses, BD FACSCanto™ II (Becton Dickinson Immunocytometry Systems, San Jose, CA) was used.

### 4.12 Collagen amount

For collagen determination, femurs of 6‐month‐old WT and miR‐146a^−/−^ mice were taken. Bone marrow was removed, and tissue was dissolved at a concentration of 100 mg/ml in 12 M HCl. Afterward, the concentration of the hydroxyprolines, and consequently, of the collagen was determined using a Total Collagen Assay Kit (QuickZyme, Leiden, Netherlands) with rat tail collagen type I as a standard, according to the manufacturer's instructions.

### 4.13 Statistical analysis

Statistical significance of two different groups was calculated using the unpaired two‐tailed Student's *t* test. All analyses were performed using GraphPad Prism 6 software. Graphs present data as mean ± *SEM*. A *p* value less than or equal to 0.05 was considered significant (*p < 0.05, **p < 0.01, ***p < 0.001).

## CONFLICT OF INTEREST

MH and JG are co‐founders and shareholders of TAmiRNA GmbH, and MH and MW are employed by TAmiRNA GmbH. All other authors have declared that no conflict of interest exists.

## AUTHOR CONTRIBUTIONS

VS, MH, JSB, BN, MT, NM, MB, and SH performed experiments; VS, MT, GH, GS, RS, GS, MW, MH, JG, JS, and SB analyzed the data; VS, JS, and SB wrote the manuscript; and all authors corrected the manuscript.

## Data Availability

The data that support the findings of this study are available from the corresponding author upon reasonable request.
