# Supplementary material for: microRNA‐146a controls age‐related bone loss
Source: Aging Cell. 2020 Oct 21;19(11):e13244. doi: 10.1111/acel.13244 (PMC7681058; doi:10.1111/acel.13244)
Supplement: Supplementary file 1 [file ACEL-19-e13244-s001.pdf]

Sup. Fig. 1

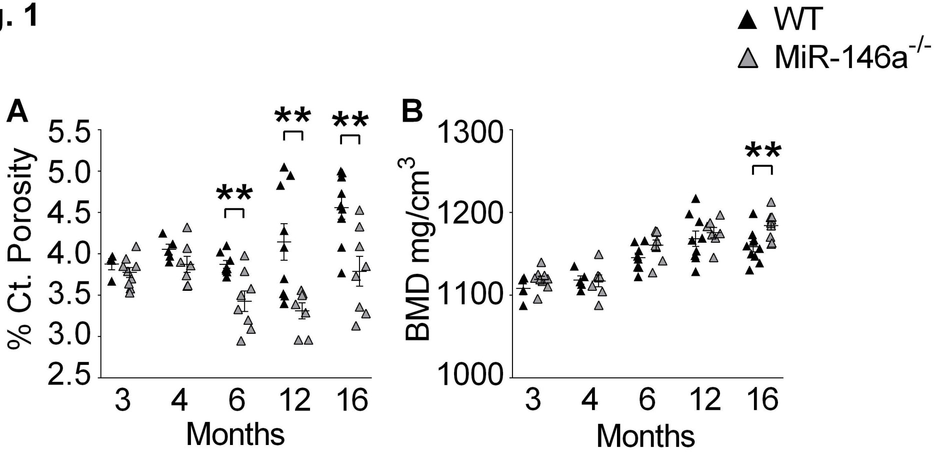

WT
MiR-146a-/-
A
% Ct. Porosity
5.5
5.0
4.5
4.0
3.5
3.0
2.5
**
**
**
3
4
6
12
16
Months
B
BMD mg/cm3
1300
1200
1100
1000
**
3
4
6
12
16
Months

Sup. Fig. 2

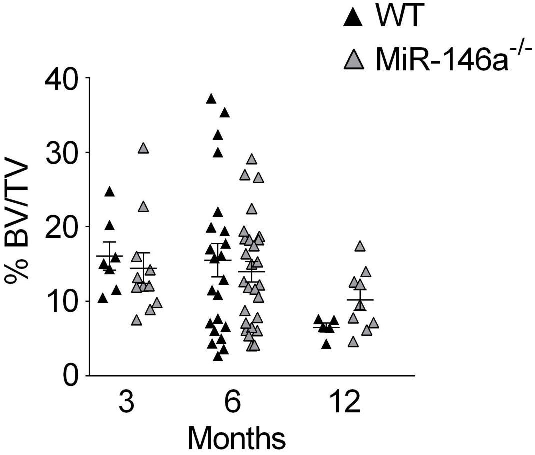

Sup. Fig. 3

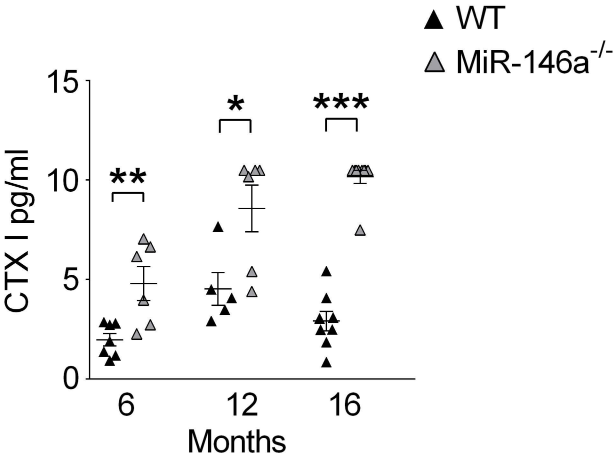

Sup. Fig. 4

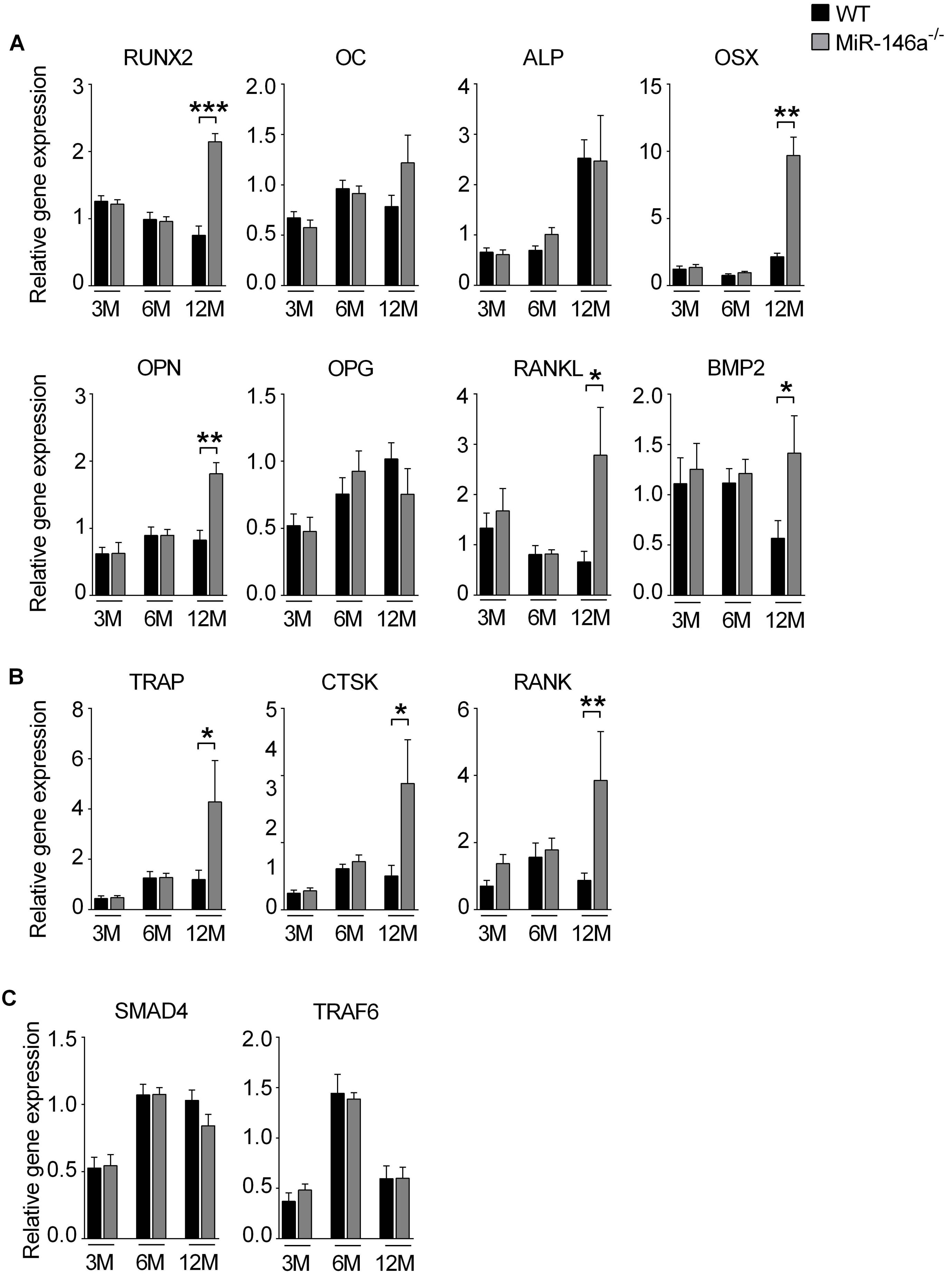
WT
MiR-146a-/-
A
RUNX2
OC
ALP
OSX
OPN
OPG
RANKL
BMP2
B
TRAP
CTSK
RANK
C
SMAD4
TRAF6
Relative gene expression
3M
6M
12M
*
**
***

Sup. Fig. 5

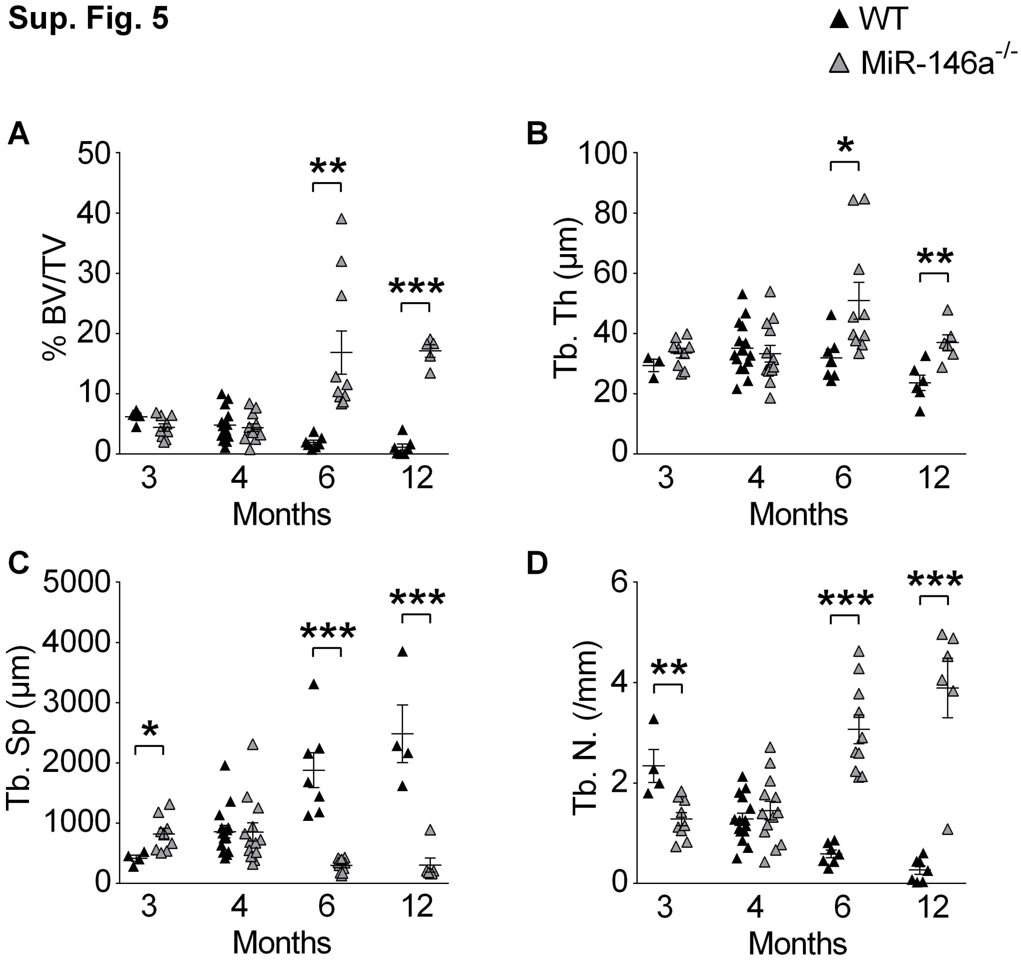

WT
MiR-146a-/-
A
% BV/TV
50
40
30
20
10
0
**
***
3
4
6
12
Months
B
Tb. Th (μm)
100
80
60
40
20
0
*
**
3
4
6
12
Months
C
Tb. Sp (μm)
5000
4000
3000
2000
1000
0
*
***
***
3
4
6
12
Months
D
Tb. N. (/mm)
6
4
2
0
**
***
***
3
4
6
12
Months

Sup. Fig. 6

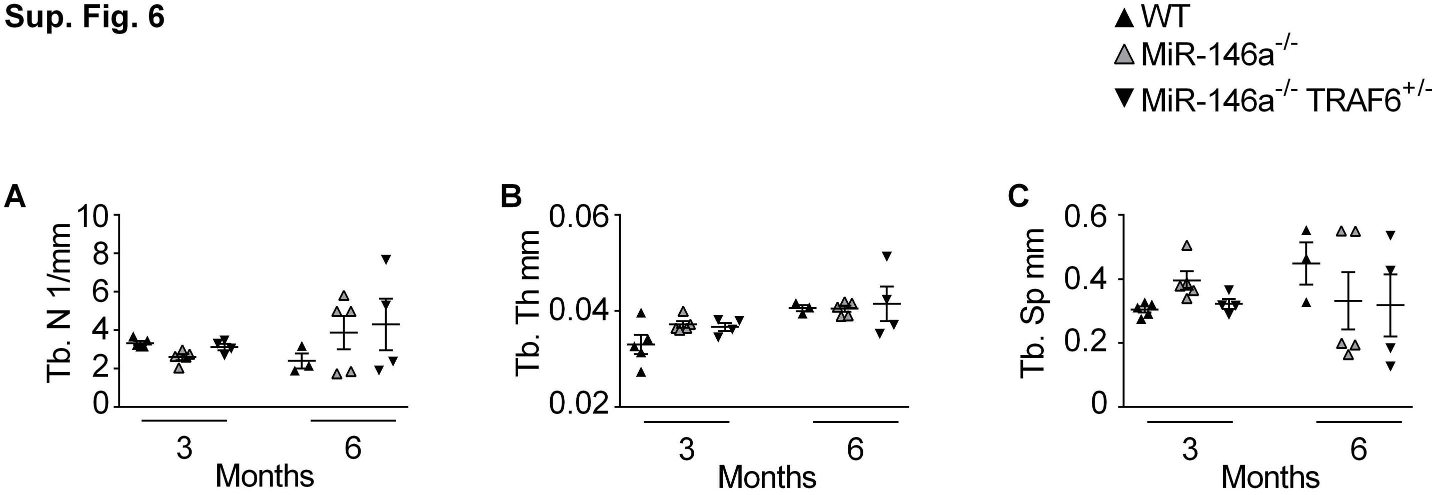

▲ WT
MiR-146a-/-
▼ MiR-146a-/- TRAF6+/-
A
Tb. N 1/mm
10
8
6
4
2
0
3
6
Months
B
Tb. Th mm
0.06
0.04
0.02
3
6
Months
C
Tb. Sp mm
0.6
0.4
0.2
0
3
6
Months

Sup. Fig. 7

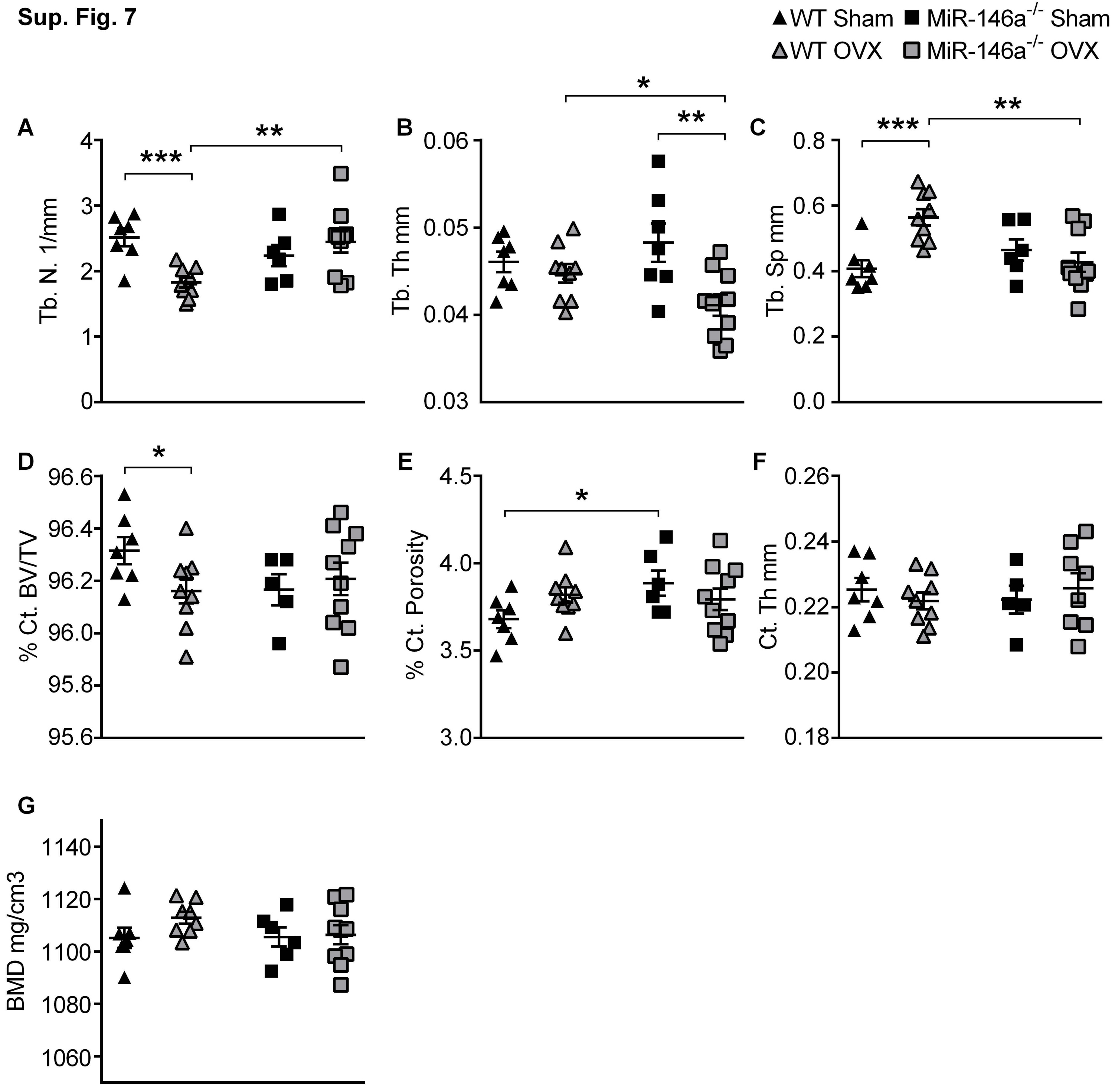
WT Sham
MiR-146a-/- Sham
WT OVX
MiR-146a-/- OVX
A
Tb. N. 1/mm
B
Tb. Th mm
C
Tb. Sp mm
D
% Ct. BV/TV
E
% Ct. Porosity
F
Ct. Th mm
G
BMD mg/cm3

Sup. Fig. 8

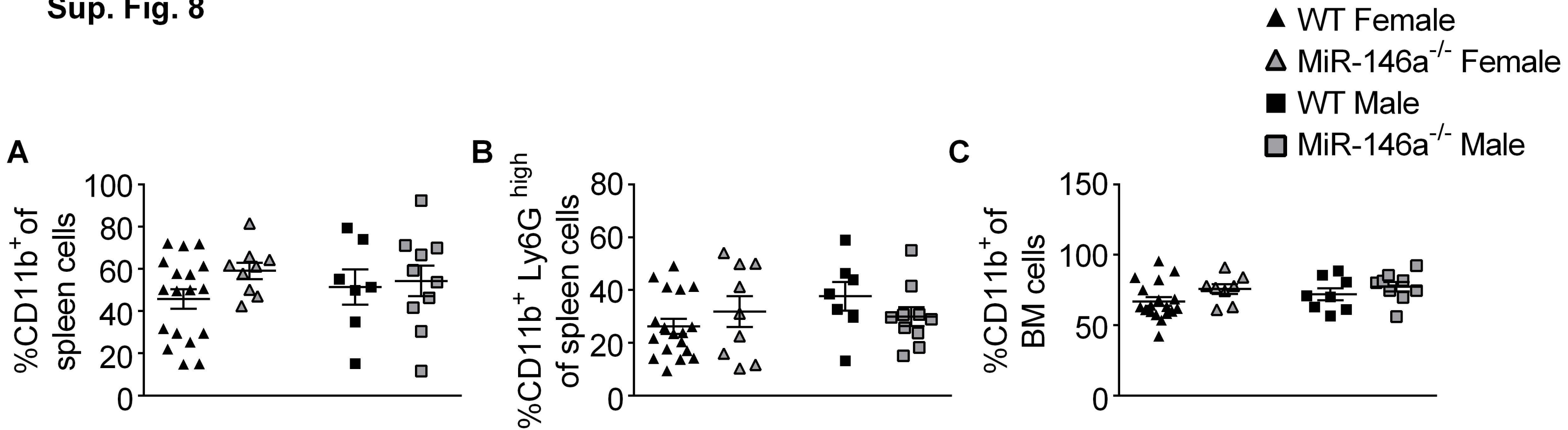

WT Female
MiR-146a-/- Female
WT Male
MiR-146a-/- Male
A
%CD11b+of spleen cells
100
80
60
40
20
0
B
%CD11b+ Ly6G high of spleen cells
80
60
40
20
0
C
%CD11b+of BM cells
150
100
50
0
